# Evaluation of a single-breath-hold radial turbo-spin-echo sequence for T2 mapping of the liver at 3T

**DOI:** 10.1007/s00330-021-08439-y

**Published:** 2021-12-23

**Authors:** Diana Bencikova, Fei Han, Stephan Kannengieser, Marcus Raudner, Sarah Poetter-Lang, Nina Bastati, Gert Reiter, Raphael Ambros, Ahmed Ba-Ssalamah, Siegfried Trattnig, Martin Krššák

**Affiliations:** 1 Department of Biomedical Imaging and Image-Guided Therapy, Medical University of Vienna, Vienna, Austria; 2 Christian Doppler Laboratory for Clinical Molecular Imaging, MOLIMA, MUW, Vienna, Austria; 3 Siemens Medical Solutions, Los Angeles, CA USA; 4 MR Application Predevelopment, Siemens Healthcare GmbH, Erlangen, Germany; 5 Research and Development, Siemens Healthcare Diagnostics GmbH, Graz, Austria; 6 Institute for Clinical Molecular MRI in the Musculoskeletal System, Karl Landsteiner Society, Vienna, Austria; 7 Division of Endocrinology and Metabolism, Department of Medicine III, Medical University of Vienna, Währinger Gürtel 18-20, A-1090 Vienna, Austria

**Keywords:** Liver, Abdomen, Magnetic resonance imaging, Breath holding, Quantitative evaluation

## Abstract

**Objectives:**

T2 mapping of the liver is a potential diagnostic tool, but conventional techniques are difficult to perform in clinical practice due to long scan time. We aimed to evaluate the accuracy of a prototype radial turbo-spin-echo (rTSE) sequence, optimized for multi-slice T2 mapping in the abdomen during one breath-hold at 3 T.

**Methods:**

A multi-sample (fat: 0–35%) agarose phantom doped with MnCl_2_ and 80 subjects (73 patients undergoing abdomen MR examination and 7 healthy volunteers) were investigated. A radial turbo-spin-echo (rTSE) sequence with and without fat suppression, a Cartesian turbo-spin-echo (Cart-TSE) sequence, and a single-voxel multi-echo STEAM spectroscopy (HISTO) were performed in phantom, and fat-suppressed rTSE and HISTO sequences were performed in in vivo measurements. Two approaches were used to sample T2 values: manually selected circular ROIs and whole liver analysis with Gaussian mixture models (GMM).

**Results:**

The rTSE-T2s values exhibited a strong correlation with Cart-TSE-T2s (*R*^2^ = 0.988) and with HISTO-T2s of water (*R*^2^ = 0.972) in phantom with an offset between rTSE and Cart-TSE maps (mean difference = 3.17 ± 1.18 ms). The application of fat suppression decreased T2 values, and the effect was directly proportional to the amount of fat. Measurements in patients yielded a linear relationship between rTSE- and HISTO-T2s (*R*^2^ = 0.546 and *R*^2^ = 0.580 for ROI and GMM, respectively).

**Conclusion:**

The fat-suppressed rTSE sequence allows for fast and accurate determination of T2 values of the liver, and appears to be suitable for further large cohort studies.

**Key Points:**

•*Radial turbo-spin-echo T2 mapping performs comparably to Cartesian TSE-T2 mapping, but an offset in values is observed in phantom measurements*.

•*Fat-suppressed radial turbo-spin-echo T2 mapping is consistent with T2 of water as assessed by MRS in phantom measurements*.

•*Fat-suppressed radial turbo-spin-echo sequence allows fast T2 mapping of the liver in a single breath-hold and is correlated with MRS-based T2 of water*.

## Introduction

Magnetic resonance (MR) has been used to non-invasively diagnose and characterize different pathological conditions of the liver. By assessing MR parameters, conditions such as steatosis [1], iron overload [2], and liver inflammation and fibrosis [3–6] can be diagnosed. Nevertheless, these conditions often manifest simultaneously; therefore, multi-parametric protocols need to be applied to obtain complex diagnosis [7, 8].

To increase patient compliance and clinical throughput, the assessment of all parameters needs to be fast. Desired elimination of breathing artifacts in the abdominal examinations underlines the need for optimized accelerated scan protocols. Whereas most of the methods from the multi-parametric protocols are fast enough (several seconds per assessment of a parameter, i.e., during single breath-hold), the data acquisition for the assessment of tissue-specific T2 relaxation times with the use of conventional multi-spin-echo techniques [9] is considerably longer. This limits the feasibility of routine T2 mapping and its incorporation in the published protocols [7, 8].

In animal models, T2 mapping was suggested to be a marker of hepatic fibrosis [10, 11], with the potential for differentiation between inflammation and fibrosis [11]. MRI T2 mapping has also been used to quantify iron content in the liver. Even though the standardized method to measure liver iron content via R2 (= 1/T2) assessment with multiple single-echo measurements is a regulatory-approved standardized method, it is a commercial proprietary solution (FerriScan®, Resonance Health), which takes several minutes to acquire data, it is restricted to 1.5 T, and there is a service fee for data analysis [12]. An alternative based on multi-echo MRS to acquire liver T2 as a simpler, time-saving method for grading of liver iron overload in two breath-holds at 1.5 T was proposed [13]. However, single-voxel spectroscopy is limited to a small portion of the liver, whereas imaging methods ideally provide information from the whole liver, assessing the spatial distribution of a parameter [13].

It has been proposed that long acquisition times and sensitivity to body motion of conventional T2 mapping can be overcome by radially sampled techniques combined with turbo-spin-echo (TSE) imaging and a tiered reconstruction [9]. Radial imaging has inherent motion insensitivity, which is due to signal averaging at the center of the k-space. Possible residual artifacts from motion and flow are spread in two directions as streaks radiating from moving structures, which makes them less severe compared to “ghosts” in Cartesian MRI. Radial TSE (rTSE) data can generate different TE-weighted images from a single k-space data set, from which a T2 map is calculated, thus being perfectly co-registered to T2w anatomical images [14]. This methodology has been already used to detect and characterize focal liver lesions [9, 15]. However, the usage of this methodology has not been extensively studied in diffuse liver disorders.

Among fibrosis and iron overload, fat accumulation is another pathology involving liver diffusely. It has been shown that fat is a confounder in T2 measurement by direct comparison of fat-suppressed and non-fat-suppressed multi-echo T2 acquisitions in iron-overloaded patients [16]. While the effect of fat suppression on R2 (1/T2) measurements in lipid-rich tissue (pancreas, vertebral bone marrow) is present, no significant effect on hepatic R2 could be shown yet [16]. Nevertheless, the conclusion of the study [16] were limited due to missing FF range of the patients included and no details on FF analysis have been reported.

Therefore, the goal of this study was to evaluate a fat-suppressed radial TSE (rTSE) sequence for accuracy of T2 mapping and feasibility in clinical protocol. The parameters were optimized for measurement in the liver at 3 T during single breath-hold, while keeping sufficient in-plane resolution and volume coverage (number of slices = 5). MRS-based T2 measurement taken as quantitative gold standard in both in vitro and in vivo conditions was complemented by Cartesian T2 mapping in phantom measurements.

### Methodological considerations of rTSE T2 mapping

T2 relaxation times are being calculated by exponential fit to multiple images with different TE_(eff)_ times. The radial sequence used here generates individual TE_(eff)_ images via echo-sharing. The general idea of echo-sharing is to use only the views with given TE in the center of the k-space, which determines the image contrast, and views at other TEs are included in the outer part of the k-space. The inclusion of the views with other TEs can be done in several ways; in this study, a “full-tier” method (which gradually includes more TE values) was used [14].

To reduce the streaking artifacts arising from T2 decay and object motion, schemes that distribute the views from different TR periods were proposed [17]. Here, a novel scheme with pseudo golden angle reordering algorithm was used [18]. The scheme is based on bit-reverse algorithm, but overcomes the main limitation of the bit-reverse scheme, which is the flexibility of the choice of echo train length (ETL). Pseudo golden angle allows for arbitrary choice of ETL, enabling shorter scan time without the compromise in image quality, which makes it suitable for clinical use.

## Materials and methods

The sequence was first tested in phantoms for its accuracy, and the effect of fat suppression on the T2 values was investigated. Finally, the sequence was evaluated in a cohort of patients and volunteers under clinical settings.

### Phantom design

The phantom was designed to investigate the influence of fat suppression in the presence of fat on the measured T2 values, and to analyze the dependence of this influence on FF. The phantom consisted of six 2% agarose samples with different fat fractions (FF) with peanut oil (0–35%) and lecithin as emulsifiers, doped with MnCl_2_ (in concentration 0.28–0.48mM) to mimic human liver in vivo T2 values (34 ± 4 ms [19]). All samples were submerged in one water container. The amount of MnCl_2_ to achieve human liver in vivo T2 values was determined in a previous separate measurement. The resulting MnCl_2_ concentration for individual samples varied due to different fat amounts, which were then filled up with MnCl_2_ solution; however the impact of this variation on resulting T2 values should be no more than ~ 10 ms based on the previous separate measurement.

### Study population

Datasets of 78 patients undergoing abdomen MR examination between October 2018 and June 2019 were selected for the study (Table 1). Seven volunteers were recruited for test-retest analysis. Written informed consent was obtained from all the participants, and the study was approved by our institutional review board.

**Table 1:** Patient characteristics

	Mean/median ± SD/IQR or *n*	Range
Patients	73	
Men	33 (45.2%)	
Women	40 (54.8%)	
Age (years)	60.6 ± 15.7	26–93
Men	62.7 ± 11.7	41–79
Women	59.2 ± 18.4	26–93
BMI (kg*m^−2^)	25.2 ± 4.2	18.5–38.1
Men	25.9 ± 4.2	18.5–37.0
Women	24.6 ± 4.2	18.8–38.1
Primary indication of MR examination Examination primary because of liver	49	
Examination primary because of pancreas	20	
Other indications	4	
Liver enzymes (*n*)*		
Bilirubin (57) (mg/dL)	0.6 ± 0.6	0.15–85.0
ASAT (58) (U/L)	27.5 ± 27.6	9–200
ALAT (62) (U/L)	50.0 ± 66.6	9–815
Gamma GT (62) (U/L)	30.0 ± 52.8	10–450
Alkaline phosphatase (62) (U/L)	65.5 ± 81.5	9–1318
Pathologies** (known/suspected or diagnosed based on the MR exam)—more than one pathology possible		
Patients with diffuse liver pathology(s)/patients with normal liver parenchyma	38/35	
Fibrosis	18	
Steatosis	21	
Hemosiderosis	8	
PBC/PSC	2/2	
Patients with liver lesion(s)/patients with no lesion	47/26	
Carcinoma	13	
Benign tumors	12	
Metastases	6	
Other lesions	16	
Patients with normal liver parenchyma and no tumor	13	

*n* = count, *measured within 6 months from the MR examination, the number in parentheses is *n* of patients with the evidence of the parameter; **known or suspected diagnoses of the patients based on the radiologic reports after the examination

### MR measurements

All measurements were performed on a 3-T MR system (MAGNETOM Prisma^fit^, Siemens Healthcare) equipped with a 64-channel body coil and a 32-channel spine coil.

A prototype rTSE sequence with pseudo golden angle reordering scheme based on prior work [18] was configured to acquire T2 maps in the abdomen within one breath-hold. The measurement parameters of rTSE sequence were adjusted to allow acquiring 5 slices of the liver within one breath-hold period: TR = 1500 ms, ETL = 29, TE range = 9.2–266.8 ms, echo-spacing = 9.2 ms, FOV 400 × 400 mm^2^, matrix size = 256 × 256, radial views = 290, FA = 180°, slice thickness = 6 mm, time of acquisition = 17 s, with fat suppression applied. T2 calculation was performed inline using a mono-exponential signal model and noise floor subtraction, and the first echo was excluded from the fit to reduce the effect of stimulated echoes.

The T2 values were compared in the phantom to those acquired with Cartesian TSE with following parameters: TR = 2630 ms, ETL = 29, TE range = 11.5–333.5 ms, echo-spacing = 11.5 ms, FOV = 400 × 400 mm^2^, matrix = 256 × 256, FA = 180°, slice thickness = 6 mm, total acquisition time = 11 min 17 s, with fat suppression applied. T2 calculation was performed inline.

To test the effect of fat suppression on T2 values, second rTSE acquisition without fat suppression was acquired in phantom. To further examine the effect of FF on the water T2 values, single-voxel multi-echo STEAM spectroscopy (HISTO, 5 TEs = 12, 24, 36, 48, and 72 ms, TR = 3 s) [20] allowing for separate water and fat signal relaxometry together with FF assessment was performed in fat samples.

The fat-suppressed rTSE-T2 map was integrated into the multi-parametric abdomen protocol targeted at patients undergoing abdomen examination at our clinic. The data for 5 slices of the T2 map were acquired across the largest partition of the liver during a 17-s breath-hold. The protocol included HISTO sequence as well, serving for FF and water T2 assessment, which were evaluated inline. The spectroscopic voxel was placed in the liver parenchyma avoiding large vessels, biliary tracts, and liver boundaries.

For the evaluation of T2 mapping variability in test-retest measurements on healthy volunteers (*n* = 7), the examination was repeated on the same examination date and the subject was taken out of the magnet and asked to sit up, and the coil was unplugged between the measurements.

### Data evaluation and analysis

The T2 values from the maps were assessed via region of interest (ROI) analysis by a single operator with 2 years of experience in liver mapping. In the phantoms, circular ROIs as large as the sample permitted, excluding the borders, were placed on the slice through the middle of the sample. In vivo liver data were evaluated with two approaches. First, the simpler approach (here referred to as “ROI analysis”) was to draw two to three circular ROIs placed in the liver parenchyma, excluding large vessels, ducts, and streaking artifacts, and to calculate T2 as the mean of the ROI values. In the second approach (referred to as “GMM analysis”), the slice with the largest liver cross-sectional coverage was selected, and a free-hand ROI encompassing the whole liver was drawn manually. Since the ROI included sections other than pure liver parenchyma, like vessels and lesions, the ROI T2 values were fit to a Gaussian mixture model implementing the expectation-maximization (EM) algorithm. The Gaussian mixture model (GMM) is a soft-clustering method, which allows a pixel to belong to more than one type of tissue with a certain probability [21, 22]. The number of components in our study was set to 2 for patients with no lesions, the first corresponding to liver parenchyma, the second to vessels. For patients with a noticeable lesion, a third component was added representing the lesion. To visually compare the GMM results, kernel density estimation (KDE) was performed to represent the distribution of T2 values in a subject. The T2 values from the subject were exported and the GMM analysis was performed in Python 3.8 with Scikit-learn [23].

### Statistical analysis

Data were presented as counts for categorical variables and as mean ± standard deviation (SD) for normally distributed variables and as median ± interquartile range (IQR) for non-normally distributed continuous variables. For the comparison of the methods and approaches, regression analysis was performed and adjusted *R* square was reported. Bland-Altman analysis was performed between radial and Cartesian acquisition in phantom, in test-retest study, and between the ROI and GMM approach. One-sample *t*-test on the differences against zero was carried out to test for statistical difference. For test-retest study, coefficients of variation (CoV) were calculated. Statistical analysis was performed in SPSS 24.0 and *p* < 0.05 was considered statistically significant.

## Results

### Phantom measurements

**Fig. 1:**
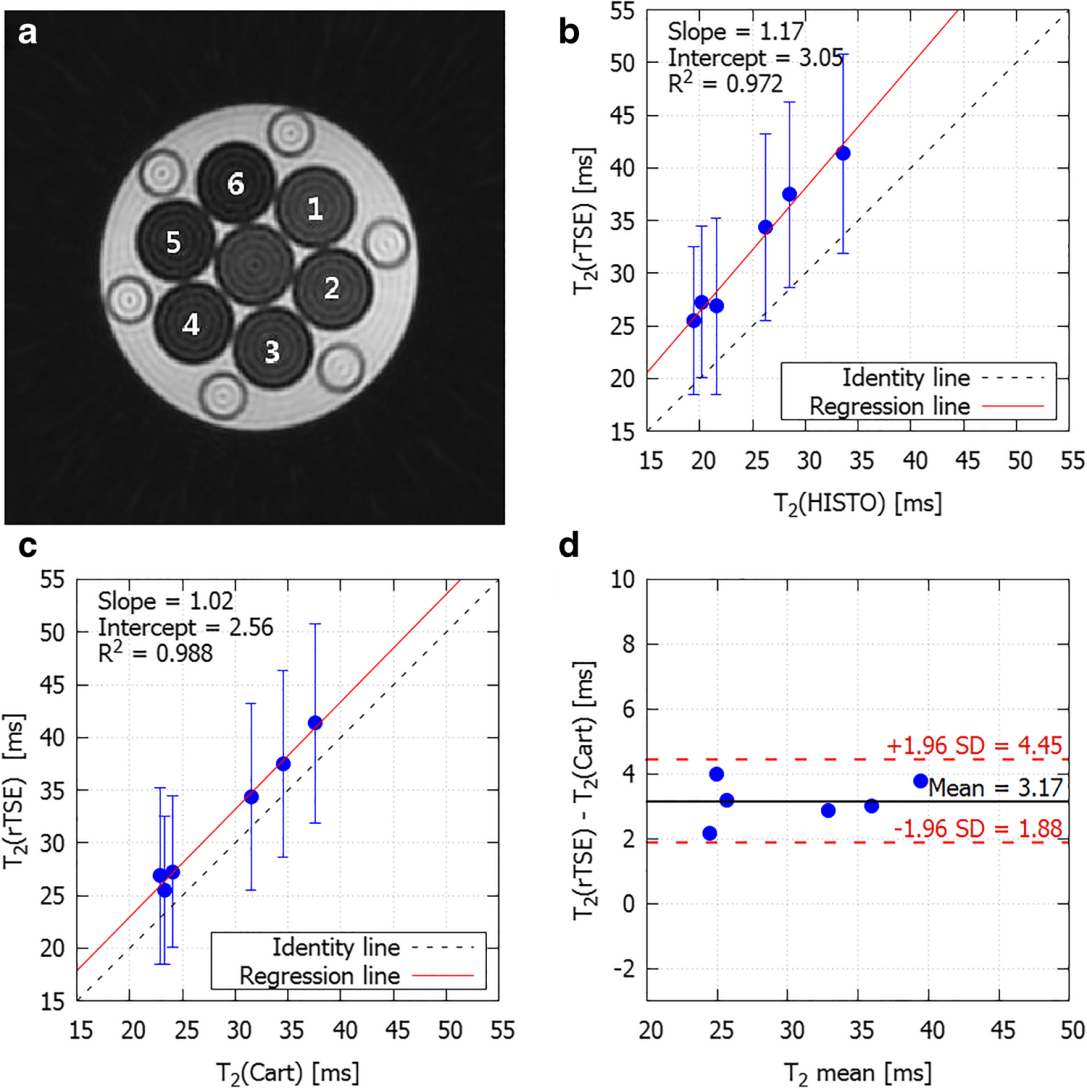
Comparison between Cartesian- and rTSE-acquired maps with the application of fat suppression: **a** representative composite image from rTSE of the multi-sample phantom. The numbers of the samples correspond with Table 2. **b** Scatterplot showing strong linear correlation between T2 values from individual samples measured by rTSE acquisitions and HISTO. **c** Scatterplot showing strong linear correlation between T2 values from individual samples measured by rTSE acquisitions and Cartesian TSE. **d** The Bland-Altman plot depicting the agreement between the two maps. The error bars represent the standard deviation of the measurements of T2 values

Table 2 summarizes all measured properties in the individual samples. The measured T2 range (22.9–37.6 ms as measured by conventional Cartesian acquisitions) in the phantom mirrors typical in vivo liver T2 values at 3 T (34 ± 4 ms [19]). A representative composite image (generated from all the views) of the phantom is shown in Fig. 1A and demonstrates sufficient quality for further analysis. Fat-suppressed rTSE-T2 exhibited a strong linear relationship with HISTO-T2 (*R*^2^ = 0.972, *p* < 0.001, Fig. 1B) with the slope 1.17 and the intercept 3.05 ms, and a strong linear relationship with fat-suppressed Cartesian-T2 (*R*^2^ = 0.988, *p* < 0.001, Fig. 1C) with the slope of 1.02 and the intercept 2.56 ms. There was a bias of 3.17 ms with upper and lower limits of agreement 4.45 and 1.88 ms between the two image-based approaches (Fig. 1D), which was statistically significantly different from zero (*p* < 0.001).

**Table 2:** Phantom properties

Sample no.	FF (%)	T2_noFS__radial (ms)	T2_FS__radial (ms)	T2_FS__cartesian (ms)	T2_HISTO_water (ms)	T2_HISTO_fat (ms)
1	0.1	40.9 ± 9.6	41.3 ± 9.4	37.6 ± 7.7	33.6	n/a*
2	3	40.4 ±8.9	37.5 ± 8.8	34.5 ±7.2	28.5	47.64
3	6.3	40.2 ± 9.7	34.3 ±8.8	31.5 ±7.7	26.2	51.39
4	13	42.2 ± 10.1	27.3 ±7.2	24.1 ±7.1	20.2	49.80
5	26.8	57.1 ±9.4	26.9 ± 8.4	22.9 ±6.8	21.5	48.54
6	36	70.4 ± 7.2	25.5 ±7.0	23.3 ±7.5	19.5	48.38

Data are presented as mean ± SD. *FF* fat fraction, *FS* fat-suppressed, *noFS* non-fat-suppressed, *insufficient amount of fat content to estimate fat T2

**Fig. 2:**
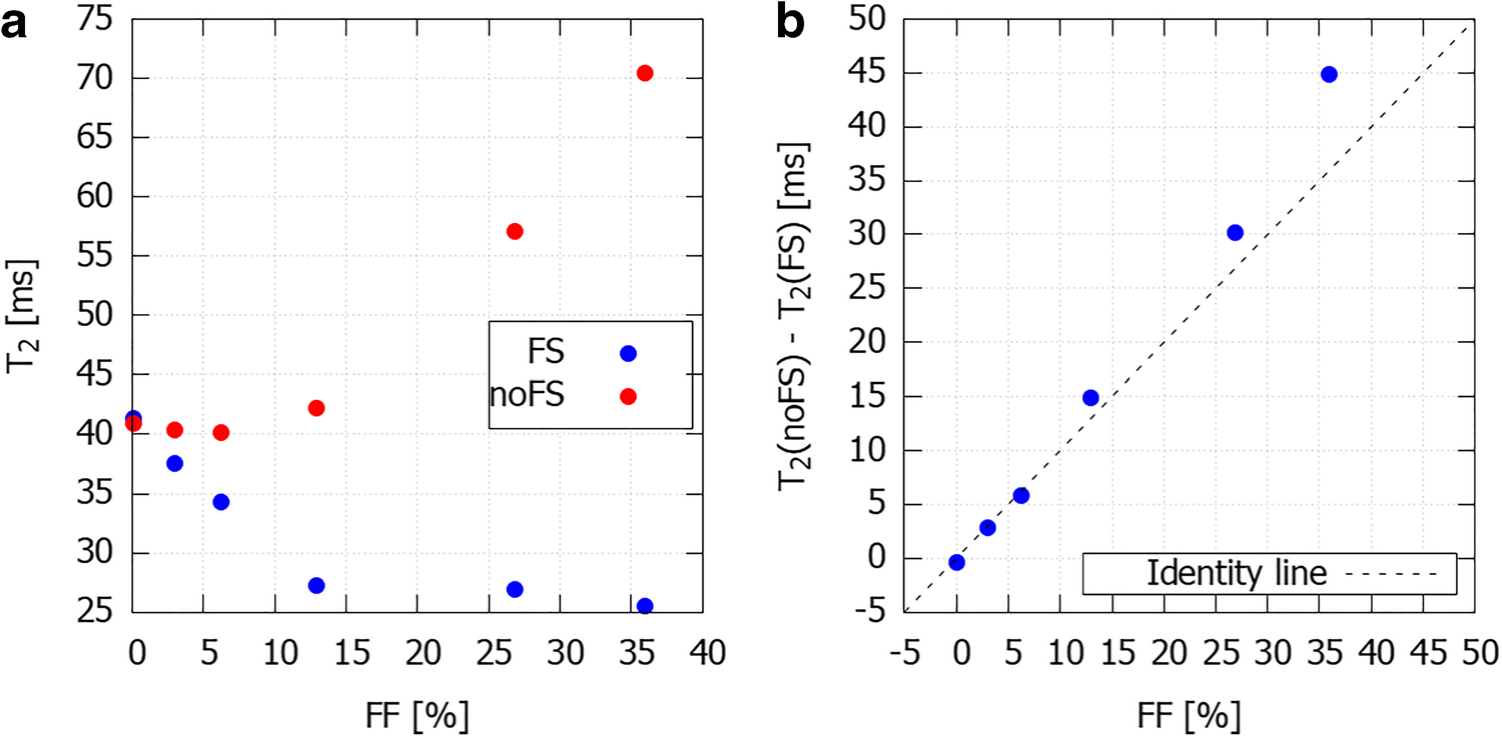
The effect of fat suppression on T2 values measured with rTSE in the phantom: **a** T2 values acquired with and without the application of fat suppression for individual samples. **b** Difference between the fat-suppressed and non-fat-suppressed acquired T2s versus fat fraction. The increase is super-linear

The HISTO FFs of the samples ranged from 0.1 to 36.0% (Table 2). The fat-suppressed and non-fat-suppressed T2 values from the rTSE sequence are shown in Fig. 2A; Fig. 2B depicts the difference between fat-suppressed and non-fat-suppressed values as a function of FF. The increase in T2 with increasing FF is super-linear in the non-fat-suppressed values in comparison to fat-suppressed values. The T2s of fat were also acquired with HISTO MRS. The non-fat-suppressed T2 values were not correlated with HISTO water T2s or HISTO fat T2s (*R*^2^ = 0.273, *p* = 0.165 and *R*^2^ = 0.170, *p* = 0.490, respectively).

### In vivo measurements

**Fig. 3:**
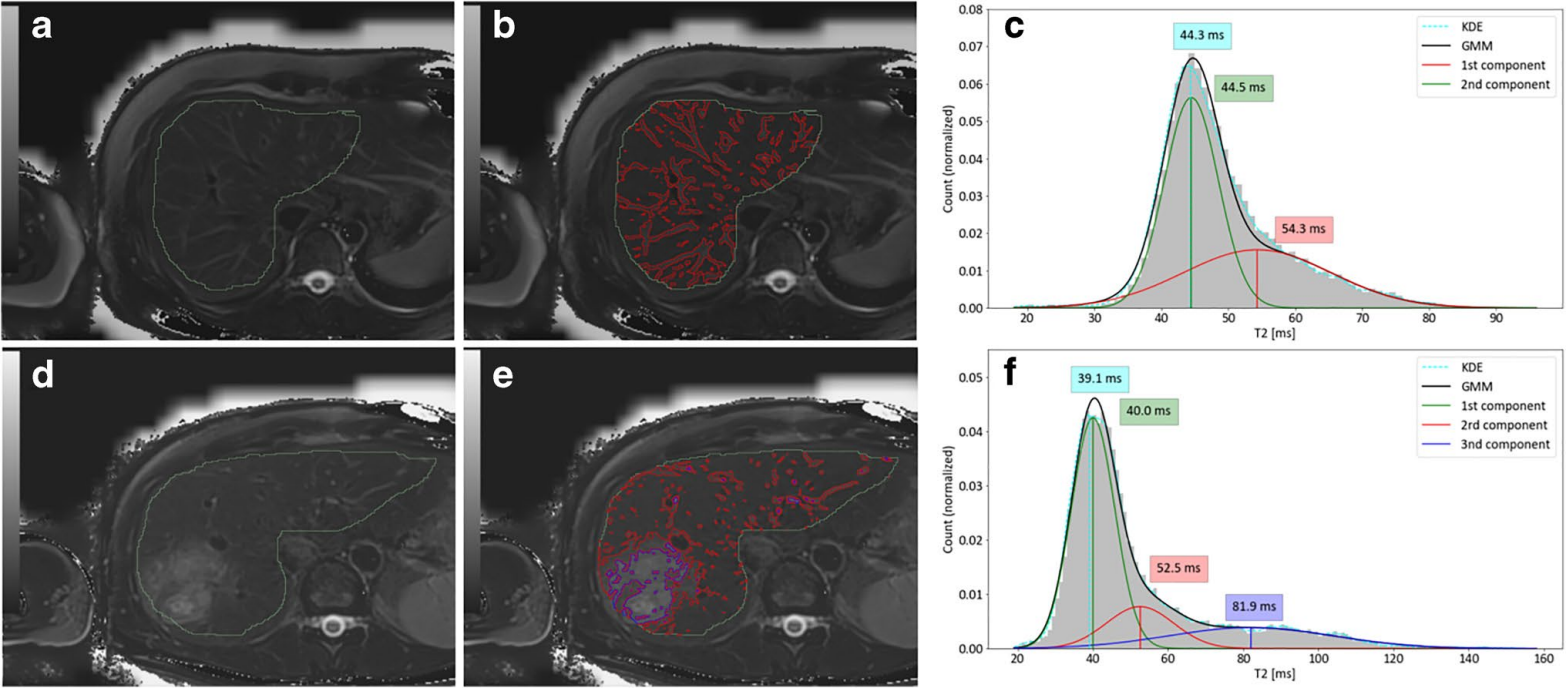
Example of the GMM analysis. 1st row: subject without a lesion, therefore 2 components were used for GMM. (**a**) Free-hand ROI delineating whole liver, (**b**) applying a threshold function on the map with the estimated mean T2 for the second component from (**c**) clearly delineates the vessels. 2nd row: subject with a lesion, therefore 3 components were used. The same procedure was applied, delineating the vessels and the tumor (**d**–**f**). Kernel density estimation (KDE) was also performed for visual comparison of the histogram to GMM. Vertical lines and the numbers represent the estimated means of the components and the peak of KDE

The exclusion criteria for the in vivo analysis were severe streaking artifacts (4) and inadequate liver coverage with rTSE (1), leaving 73 patients to evaluate. Clinical characteristics of patients and summary of primary indications for MR exams are given in Table 1. Table 3 summarizes the FF and T2 values measured by HISTO and rTSE fat-suppressed acquisition, analyzed with both approaches (ROI and GMM), and Fig. 3 depicts an example of the GMM analysis.

**Table 3:** FF and T2 values in patients and volunteers

	FF (HISTO) (%)		T2 (HISTO) (ms)		T2 (rTSE_ROI) (ms)		T2 (rTSE_GMM) (ms)	
	Mean ± SD	(Range)	Mean ± SD	(Range)	Mean ± SD	(Range)	Mean ± SD	(Range)
Patients	4.7 ± 5.4	(0.5–32.0)	27.1 ± 4.6	(18.6–45.9)	38.7 ± 6.8	(23.8–58.3)	38.4 ± 6.0	(23.6–52.9)
Volunteers	2.0 ± 1.6	(0.7–5.2)	27.6 ± 3.1	(22.2–31.7)	41.2 ± 5.1	(34.7–48.2)	39.3 ± 3.8	(34.5–44.7)

Values for volunteers are calculated as mean from both measurements. *FF* fat fraction, *SD* standard deviation, *rTSE* radial turbo-spin-echo, *ROI* region of interest, *GMM* Gaussian mixture model

**Fig. 4:**
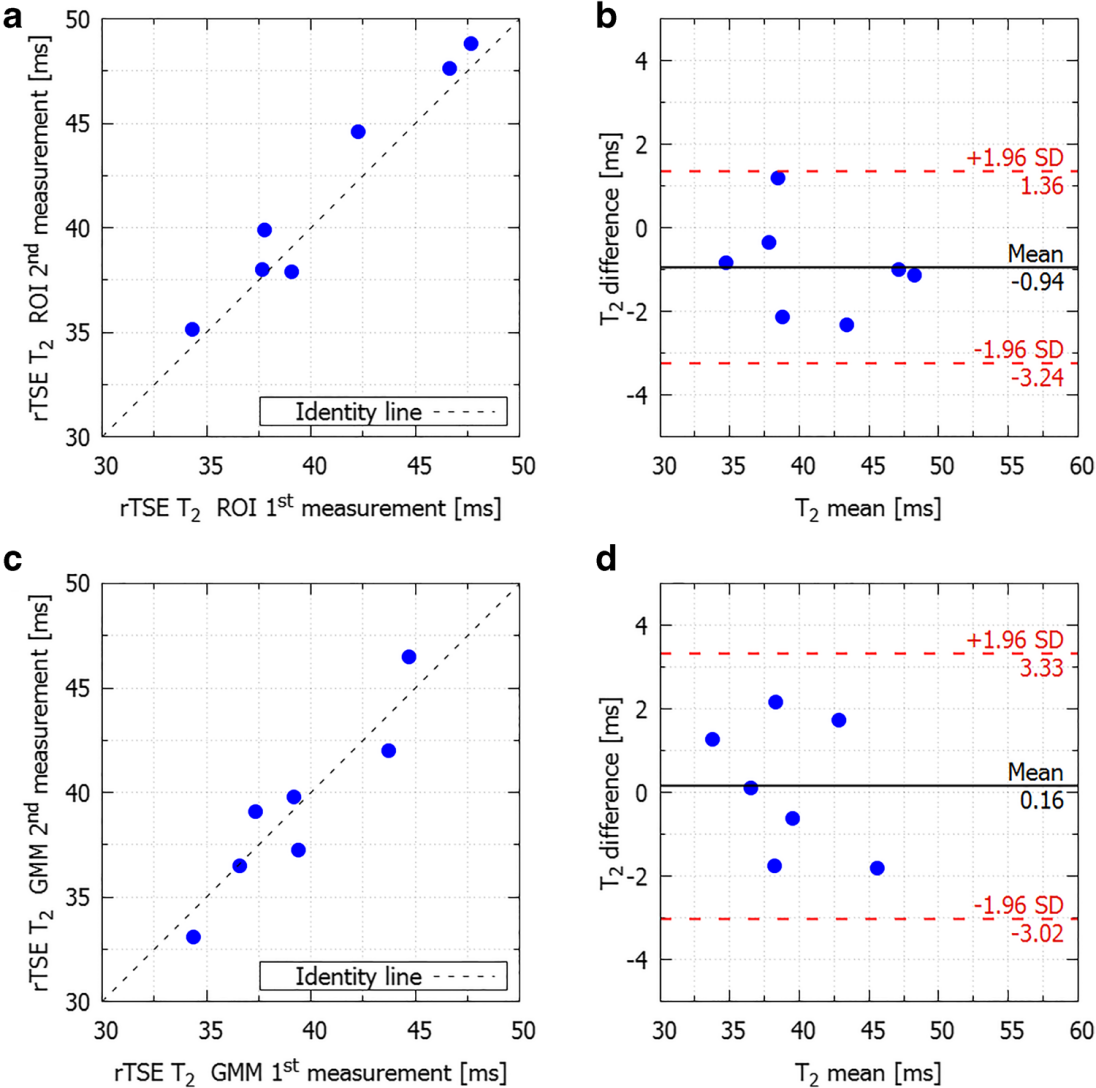
Scatterplot showing linear correlation and Bland-Altman analysis between the 1^st^ and 2^nd^ measurement of the test-retest study for ROI approach (**a** and **b**) and GMM approach (**c** and **d**)

For the test-retest measurements in volunteers, the T2 values for both approaches strongly correlated with *R*^2^ = 0.943 (*p* < 0.001) for ROI analysis and *R*^2^ = 0.831 (*p* = 0.003) for GMM analysis with small differences that were not statistically significantly different from zero (mean difference between the measurements was 0.94 ms with upper and lower limits of agreement 1.36 ms and − 3.24 ms for ROI analysis, *p* = 0.079, and mean difference 0.16 ms with upper and lower limits of agreement 3.33 ms and − 3.02ms for GMM analysis, *p* = 0.806, Fig. 4). The CoV was 2.18% for ROI analysis and 2.40% for GMM analysis.

**Fig. 5:**
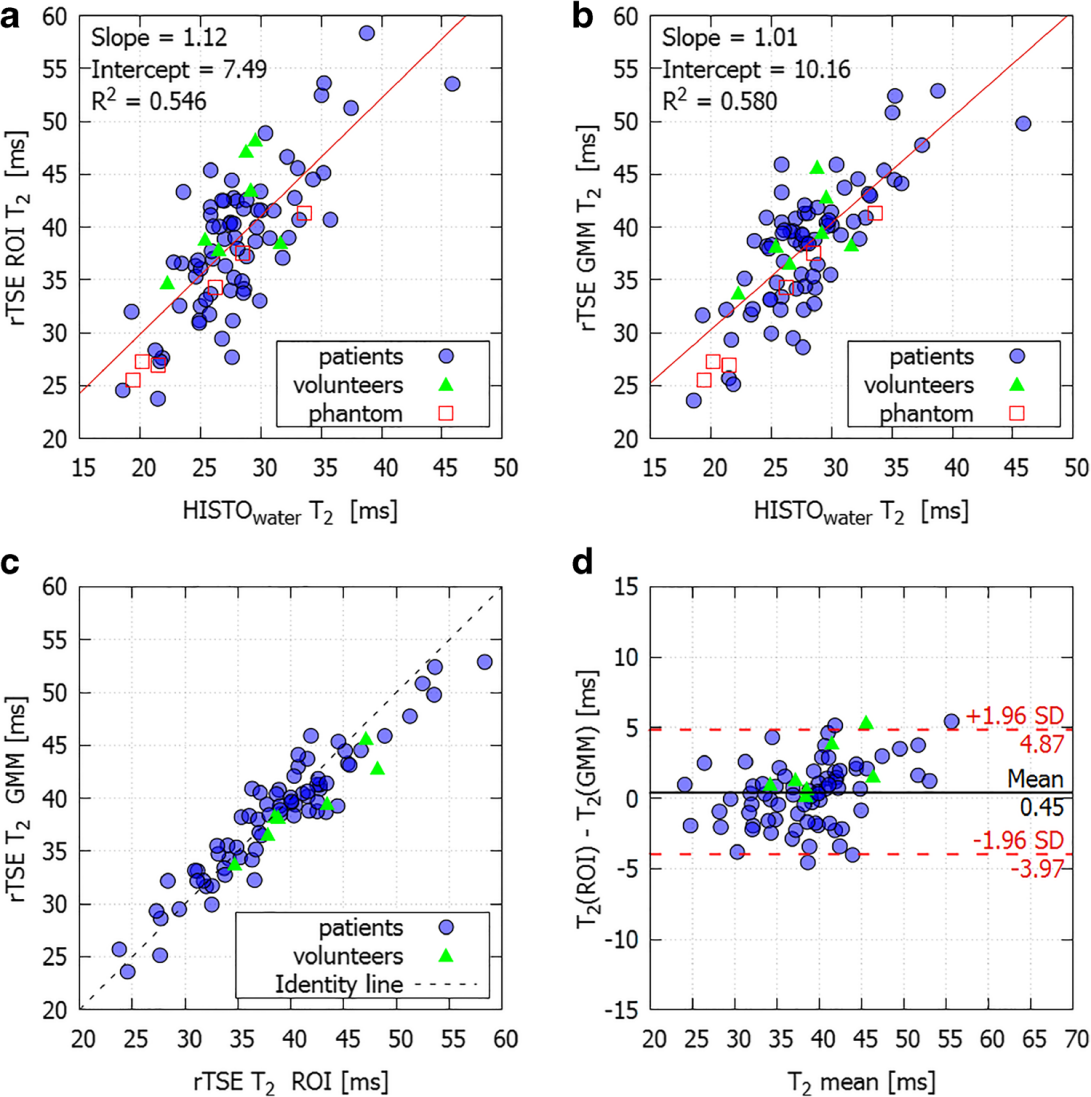
Scatterplot showing linear correlation between T2 values measured by HISTO and fat-suppressed rTSE analyzed with (**a**) ROI approach and (**b**) GMM approach in patients and volunteers. The phantom data are shown as well, but are not included in the regression analysis. (**c**) Scatterplot showing linear correlation and (**d**) Bland-Altman plot showing agreement between the two approaches of analyzing the rTSE T2 maps

For the combined in vivo measurements, HISTO- and rTSE-T2 exhibited a linear relationship in both cases (*R*^2^ = 0.546 and *R*^2^ = 0.580 for ROI and GMM approach, respectively) with a slope of 1.12 (*p* < 0.001) and intercept 7.49 ms (*p* = 0.32) for ROI analysis and with a slope of 1.01 (*p* < 0.001) and intercept 10.16 ms (*p* < 0.001) for GMM analysis (Fig. 5A and B). The results from both approaches highly correlated (*R*^2^ = 0.894, *p* < 0.001) and were in a good agreement with mean difference of 0.45 ms that was not statistically significant (*p* = 0.079), and upper and lower limits of agreement 4.87 and − 3.97 (Fig. 5C and D).

## Discussion

The co-existent pathological changes in the liver may interact and mutually confound the quantification methods. Therefore, multi-parametric protocols are required to assess the pathologic parameters and control the confounders. In this paper, we have demonstrated the feasibility of performing a T2 mapping measurement in the liver during single breath-hold making it suitable for inclusion in larger multi-parametric research and clinical protocols.

While the STEAM-T2 values agree with previously published results in NAFLD population [24, 25], and while we could detect excellent correlation between radial- and Cartesian-based T2 values in phantom measurement, we could reveal an absolute offset between these two approaches. Depending on the clinically required accuracy, this may mean that results from the current state of the rTSE and Cartesian methods cannot be used interchangeably or directly compared. The root cause of the offset remains to be investigated; potential candidates are the different TEs used, and details of the radial acquisition and tiered view-sharing [16] reconstruction.

Our phantom experiments suggest that fat suppression markedly influences the T2 value. With non-fat-suppressed acquisition, non-chemical-shift selective T2 decay is assessed; therefore, relaxation properties of two main components, water and fat, are intrinsically combined. Lipids have longer T2 relaxation times than water [20, 24]; therefore, resulting overall T2 in mixed acquisition is longer than T2 of water signal alone. With the application of fat suppression, the fat signal contribution in the resulting T2 is reduced, if not eliminated. This was confirmed in the phantom measurements, where fat-suppressed rTSE T2 values correlated perfectly with HISTO-water T2 values, while there was no correlation with HISTO without application of fat suppression. Papakonstantinou et al [16] reported differences in R2 mapping between fat-suppressed and non-fat-suppressed acquisitions, but direct comparison with this paper is not possible, because no FF of the organs, nor the dependence on FF level was given. With radial acquisition, fat suppression has the additional benefit of reducing the streaking artifacts that could arise from subcutaneous adipose tissue near the surface coils. For this reason, only fat-suppressed T2 maps were acquired and analyzed in vivo.

We used two approaches to obtain T2 values from the maps. The ROI method is the most widely used in clinical practice, but is subjective to observer variability. The GMM method is less observer-dependent but requires additional post-processing. Clark et al [26, 27] used fitting multiple Gaussians to R2 distribution of the liver and was able to distinguish different tissue types. However, they did not describe the fitting procedure in detail; here, GMM employing E-M algorithm implemented in Scikit-learn was used [23]. We have shown that both methods yielded comparable results, which indicates that the simple ROI method applied rigorously by a single experienced observer performs well, and the results are not biased.

Spectroscopically obtained R2s were compared to FerriScan-R2 (1/T2s) for the iron assessment in the liver [13]. Regression analysis between the FerrsiScan-R2 and HISTOV-R2 (variant of HISTO adapted for high iron levels) yielded a non-unit regression slope but showed good correlation (*R*^2^ = 0.889). Note that the analyzed range of R2 (1/T2) values was much broader than in our study, which might be the source of stronger correlation compared to our study (*R*^2^ = 0.889 vs 0.546/580 corresponding to [13] vs ROI/GMM approach in our study, respectively).

Some authors studied liver T2s as a marker of disease using only dual-echo TSE sequences, which introduces lower statistical certainty in the fitting algorithm [11]. Despite this limitation, they found promising outcomes with staging the liver inflammation and fibrosis grade with T2s. As the rTSE method is capable of sampling the full decay curve, it might allow more accurate characterization of diffuse liver disorders.

As the limitations of our study, we should certainly name: (i) missing of direct comparison to a “gold standard” for in vivo liver T2 mapping, and it is yet unclear which method that could be. The theoretical equivalence of spectroscopy (HISTO) and imaging has not been established yet, and has to be further investigated [13]. (ii) The role of signal model and fit algorithm details in combination with both the radial fast spin-echo nature of the acquisition was not investigated further in vivo. The widespread approach to discard first echo and to apply noise floor subtraction was used [28]. (iii) We also did not compare the possible effects of different sequence parameterizations like ETL and FOV on T2 and streaking artifacts, which was beyond the scope of the study.

Indirect (stimulated) echoes can confound T2 values, and approaches to correct for this have been proposed, also for estimation of T2s from highly under-sampled data [29]. This correction has meanwhile been integrated into newer prototype versions of inline processing, together with novel de-streak algorithms, and will be evaluated in near future. To help estimating the accuracy of any given fit, a measure of model consistency should be integrated into the inline implementation [30].

In conclusion, the radial T2 mapping with short acquisition times, thus enabling breath-hold acquisition, presented here compares favorably to other methods both in phantoms and in vivo, works reliably in a clinical protocol, and appears to be suitable for further studies of diffuse liver disorders and to be a part of multi-parametric protocols.

## Abbreviations

ALAT: Alanine aminotransferase
ASAT: Aspartate aminotransferase
BMI: Body mass index
EM: Expectation-maximization
ETL: Echo train length
FA: Flip angle
FF: Fat fraction
FOV: Field of view
GMM: Gaussian mixture model
KDE: Kernel density estimation
MRS: Magnetic resonance spectroscopy
PBC: Primary biliary cirrhosis
PSC: Primary sclerosing cholangitis
ROI: Region of interest
TE: Echo time
TR: Repetition time
TSE: Turbo-spin-echo

## Declarations

### Guarantor

The scientific guarantor of this publication is Martin Krššák, PhD.

### Statistics and biometry

No complex statistical methods were necessary for this paper.

### Study subjects or cohorts overlap

Some study subjects or cohorts have been previously reported in 36^th^ Annual conference of ESMRMB, Rotterdam, 2019, and 28^th^ Annual meeting of ISMRM in Sydney/Paris, 2020.

### Methodology

•prospective

•experime

•performed at one institution
